# Severe but not mild hypercapnia affects the outcome in patients with severe cardiogenic pulmonary edema treated by non-invasive ventilation

**DOI:** 10.1186/s13613-015-0055-y

**Published:** 2015-06-07

**Authors:** Damien Contou, Chiara Fragnoli, Ana Córdoba-Izquierdo, Florence Boissier, Christian Brun-Buisson, Arnaud W Thille

**Affiliations:** AP-HP, Réanimation Médicale, Groupe Hospitalier Henri Mondor - Albert Chenevier, Créteil, France; Réanimation Médicale, CHU de Poitiers, 2 rue de la Milétrie, 86021 Poitiers, France; INSERM CIC 1402 (ALIVE group), Université de Poitiers, Poitiers, France

**Keywords:** Intensive care unit, Acute respiratory failure, Cardiogenic pulmonary edema, Non-invasive ventilation, Hypercapnia, Respiratory acidosis

## Abstract

**Background:**

Patients with severe cardiogenic pulmonary edema (CPE) are frequently hypercapnic, possibly because of associated underlying chronic lung disease (CLD). Since hypercapnia has been associated with outcome, we aimed to identify factors associated to hypercapnia and its role on outcome of patients with CPE and no underlying CLD.

**Methods:**

Observational cohort study using data prospectively collected over a 3-year period. After excluding patients with any CLD or obstructive sleep apneas, all patients treated by non-invasive ventilation (NIV) for severe CPE were included. Hypercapnia was defined as PaCO_2_ >45 mmHg and non-rapid favorable outcome was defined as the need for intubation or continuation of NIV for more than 48 h.

**Results:**

After excluding 60 patients with underlying CLD or sleep apneas, 112 patients were studied. The rates of intubation and of prolonged NIV were 6.3 % (*n* = 7) and 21.4 % (*n* = 24), respectively. Half of the patients (*n* = 56) had hypercapnia upon admission. Hypercapnic patients were older, more frequently obese, and were more likely to have a respiratory tract infection than non-hypercapnic patients. Hypercapnia had no influence on intubation rate or the need for prolonged NIV. However, patients with severe hypercapnia (PaCO_2_ >60 mmHg) needed longer durations of NIV and intensive care unit (ICU) stay than the others.

**Conclusions:**

Among the patients admitted for severe CPE without CLD, half of them had hypercapnia at admission. Hypercapnic patients were older and more frequently obese but their outcome was similar compared to non-hypercapnic patients. Patients with severe hypercapnia needed longer durations of NIV than the others without increase in intubation rate.

## Background

Cardiogenic pulmonary edema (CPE) is usually rapidly reversible using appropriate medical therapy including high dose of vasodilators and diuretics [1]. The use of non-invasive ventilation (NIV) induces a more rapid improvement in respiratory distress than does standard oxygen therapy [2] and reduces the need for intubation and mortality of the most severe patients [3–5], with less than 10 % of patients with severe CPE treated with NIV needing intubation [2, 6–12].

Hypercapnia is common in patients with severe CPE [13]. At NIV initiation, around 20 % to 50 % of patients with severe CPE are hypercapnic, as defined as a PaCO_2_ >45 mmHg [6–12]. Hypercapnia is considered as a marker of severity and has been associated with a higher risk of intubation [14]. Several controlled clinical trials have found that hypercapnia was associated with a poor outcome despite the use of NIV [6, 8]. Factors associated with hypercapnia during CPE are poorly understood, although many patients with CPE may have an associated underlying chronic lung disease (CLD) promoting hypercapnia. Although some studies excluded patients with a known severe airflow obstruction [6, 8, 12], some patients may still have non-documented mild or moderate chronic obstructive pulmonary disease (COPD), obesity-hypoventilation syndrome, obstructive sleep apneas, or rib cage abnormalities. The combination of left heart dysfunction and chronic lung disease is common; in a large clinical trial assessing NIV in more than 1000 patients with CPE [2], nearly 20 % of them had an underlying CLD. Conversely, in patients admitted in intensive care unit (ICU) for acute exacerbation of COPD, acute left ventricular dysfunction is identified as the main reason for acute respiratory failure in more than 40 % of the cases [15]. It is well demonstrated that NIV markedly reduces intubation and mortality rates in hypercapnic patients with exacerbation of COPD [16–18]. However, the overall rate of NIV failure in patients with acute-on-chronic lung disease is significantly higher than in those admitted for severe CPE [19] with an intubation rate reaching 25 to 30 % in recent surveys [20, 21].

We therefore conducted this study with the aims (1) to assess the incidence and factors associated with hypercapnia in patients treated by NIV for severe CPE, after excluding all those with any suspected underlying CLD, and (2) to assess the influence of hypercapnia on outcome.

## Methods

The study was conducted in the 24-bed medical ICU at Henri Mondor University hospital in Créteil, France. The study was approved by the Institutional Review Board of the French Society for Respiratory Medicine.

### Inclusion of patients with CPE

During a 3-year period (from June 2008 to June 2011), data on all consecutive patients receiving NIV as initial ventilatory support for acute respiratory failure were included, as previously described [19, 22]. Acute respiratory failure was defined as recent dyspnea with a respiratory rate >25 breaths/min and/or sternocleidomastoid muscle activation and/or hypoxemia (defined as a SpO_2_ below 90 % while breathing room air). The diagnosis of CPE was defined as an acute respiratory failure in a patient with all of the following criteria: a compatible history of prior CPE or chronic heart failure, clinical signs of left and/or right cardiac failure, increase in NT-proBNP above 1000 pg/ml, bilateral alveolar and/or interstitial opacities on chest X-ray, and increase in left ventricular filling pressure on echocardiography indicated by a mitral E/A velocity ratio >2 using PW Doppler or E/e’ velocity >15 cm/s using tissue Doppler [23], in the absence of pneumonia. Hypercapnia was defined according to the literature [6–12] as a PaCO_2_ above 45 mmHg. All patients with CPE received concomitant standard medical therapy including vasodilators (repeated boluses of IV isosorbide-dinitrate) and diuretics (at least 80 mg of furosemide).

### Exclusion of patients with chronic lung disease

The main reason that led to NIV initiation was systematically recorded by the physician in charge and all patients receiving NIV as initial ventilatory support for acute respiratory failure were then stratified into two subgroups according to their PaCO_2_ at admission: (1) hypercapnic patients with a PaCO_2_ >45 mmHg [19], and (2) hypoxemic non-hypercapnic patients with a PaCO_2_ ≤45 mmHg [22]. For the purpose of the current study, all patients with CPE and documented chronic lung disease were secondarily excluded. An independent pulmonologist (ACI) reviewed all medical charts to exclude patients having any underlying chronic lung disease (CLD), i.e., those with chronic obstructive pulmonary disease, obesity-hypoventilation syndrome, obstructive sleep apnea syndrome (OSA), or another reason for chronic respiratory failure. COPD was suspected on history of smoking, symptoms of chronic bronchitis, dyspnea, and/or chronic hypercapnia, and/or emphysema on chest radiograph or CT scanner. Obesity-hypoventilation syndrome was defined as obesity with a body mass index >30 kg/m^2^, chronic hypercapnia with a PaCO_2_ >45 mmHg, in the absence of airflow obstruction using spirometry. Obstructive sleep apneas was defined as apnea-hypopnea index above 10/h using polysomnography or clinical symptoms using Epworth scale [24], associated with episodes of nocturnal desaturation during their ICU stay. Before and during the study period, several prospective physiological studies have been conducted in our unit to investigate sleep quality using polysomnography in patients admitted for acute hypercapnic respiratory failure [25, 26]. Therefore, special attention was given to identify patients with sleep apneas and polysomnography was usually done before discharge in those with nocturnal desaturation. Hypercapnic patients admitted with acute respiratory failure without documented lung disease were systematically screened and seen 3 months later to perform polysomnography, physiological respiratory tests, thoracic CT scan, and echocardiography, and those having secondarily documented chronic lung disease or airflow obstruction were excluded from this study.

### Non-invasive ventilation protocol

The study was conducted after the implementation, in June 2008, of a nurse-driven NIV protocol which included prospective daily collection of clinical data and ventilatory parameters on a specific NIV monitoring form [19, 22]. NIV was delivered in pressure-support ventilation (PSV) mode with an ICU ventilator using a dedicated NIV mode (Evita XL, Dräger, Lübeck, Germany, or Engström Carestation, GE Healthcare, Fairfield, CT, USA). NIV was started using a PS level of 8 cm H_2_O and a positive end-expiratory pressure (PEEP) level of 5 cm H_2_O. PSV was gradually increased by 2 cm H_2_O steps to reach a targeted expiratory tidal volume around 6–8 ml/kg predicted body weight and FiO_2_ was gradually adjusted by 5 % step to reach targeted SpO_2_ ≥94 %. Non-invasive ventilation was applied intermittently for periods of at least 2 h, with a minimal duration of 6 h per day, or continuously in case of severe hypoxemia, and was maintained until signs of respiratory distress improved. NIV was delivered via a non-vented full-face mask (Free Motion™ RT041, Fisher and Paykel, Auckland, New Zealand or Ultra Mirage™, Resmed, CA, USA). An algorithm was used by nurses in case of leaks, which involved first repositioning of the mask, then reducing the PEEP level at 2 cm H_2_O, third, reducing the pressure-support level by steps of 2 cm H_2_O until the minimal expiratory volume was reached, and fourth changing the mask interface. A mobile cart containing all types and sizes of interfaces was available at the bedside during initiation of NIV.

### Data collection and definitions

From the NIV monitoring forms, we analyzed the number and duration of NIV sessions, ventilator settings (pressure-support level, positive end-expiratory pressure, FiO_2_), ventilatory parameters (SpO_2_, respiratory rate, expiratory tidal volume), level of consciousness assessed using the Richmond Agitation-Sedation Scale [27], NIV tolerance, amount of leaks, and hemodynamic parameters (heart rate, blood pressure). Blood gases were routinely measured 1 h after initiation of NIV.

Since CPE is usually characterized by a rapid improvement in respiratory distress within the first hours of therapy, a non-rapid favorable outcome was defined as the need for intubation or for prolonged NIV for more than 48 h. The following criteria were used for tracheal intubation: hypercapnic coma, psychomotor agitation making nursing care impossible and requiring sedation, frank worsening in signs of respiratory distress with a respiratory rate above 40 breaths/min under NIV, SpO_2_ remaining below 90 % despite FiO_2_ 100 %, and persistent hypotension. Worsening respiratory acidosis or absolute values of pH/PCO_2_ were not used as criteria for intubation in the absence of other signs cited above.

### Statistical analysis

All data are expressed as mean ± standard deviation (±SD) or as median and [25^th^–75^th^] percentiles, and dichotomous variables are reported as number and percentage (%). Qualitative data were compared using the Fisher’s exact test, and quantitative data using the Mann–Whitney non-parametric test. To evaluate independent factors associated with non-rapid favorable outcome, we performed a logistic regression analysis using a backward procedure including in the model all non-redundant variables associated with prolonged NIV or intubation with a *p* value <0.10. We considered two-tailed *p* values <0.05 as significant. Statistical analyses were performed using the statistical software package STATA version 13.1 (STATA Corp., College Station, TX, USA).

## Results

### Patients

**Fig. 1:**
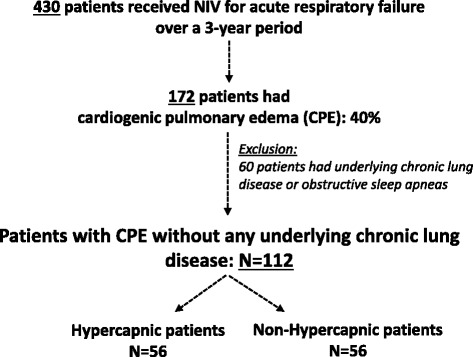
Flow chart of patients included in this study

Over a 3-year period, 172 patients received NIV for severe CPE. After excluding 60 patients with associated CLD, 112 patients were retained in the analysis (Fig. 1).

The main precipitating factor for CPE was rapid atrial fibrillation in 9.8 % of the cases (*n* = 11), acute coronary syndrome in 7.1 % (*n* = 8), hypertensive crisis in 12.5 % (*n* = 14), fluid overload in 23.2 % (*n* = 26), respiratory tract infection in 12.5 % (*n* = 14), extra-pulmonary sepsis in 7.1 % (*n* = 8), valvular disease in 4.5 % (*n* = 5), and auricular-ventricular block in 1.7 % (*n* = 2); no precipitating factor was identified in the remaining 24 (21.4 %) patients.

Overall, the rates of intubation (all in the first 24 h) and of prolonged NIV (>48 h) were 6.3 % (*n* = 7) and 21.4 % (*n* = 24), respectively, for a total rate of “non-rapid favorable outcome” of 27.7 % (*n* = 31). Overall in-ICU mortality was 4 % (4 of the 7 patients who needed intubation, 57 %).

### Characteristics of hypercapnic patients

Comparisons between hypercapnic patients and non-hypercapnic patients are shown in Table 1. Hypercapnic patients were older, more frequently obese, and were more likely to have a respiratory tract infection than non-hypercapnic patients. Despite higher pressure-support levels, hypercapnic patients had lower expiratory tidal volume and lower minute ventilation under NIV and had more frequently acidosis and altered mental status. By contrast, non-hypercapnic patients were more hypoxemic and had more frequently tachycardia at admission than hypercapnic patients. Outcomes did not differ between hypercapnic and non-hypercapnic patients (Table 1).

**Table 1:** Comparison of patients according to the presence or not of hypercapnia at admission

	Non-hypercapnic, *N* = 56	Hypercapnic, *N* = 56	*P* value
Characteristics of the patients			
Age, years	68.7 ± 14.0	76.6 ± 11.8	<0.01
Male gender, *n* (%)	26 (46 %)	30 (54 %)	0.71
SAPS II, points	36.5 ± 12.5	39.1 ± 14.1	0.38
BMI, kg/m^2^	24.7 ± 6.1	27.0 ± 5.6	0.03
BMI >30 kg/m^2^, *n* (%)	4/47 (9 %)	14/45 (31 %)	<0.01
History of smoking, *n* (%)	16 (29 %)	24 (43 %)	0.17
Underlying ischemic cardiopathy, *n* (%)	30 (54 %)	25 (45 %)	0.35
Baseline LVEF, %	41 ± 15	42 ± 12	0.84
Reason for CPE			
Atrial fibrillation, *n* (%)	7 (12.5)	4 (7.1 %)	0.52
Acute myocardial infarction, *n* (%)	3 (5.4 %)	5 (8.9 %)	0.71
Hypertensive crisis, *n* (%)	8 (14.3)	6 (10.7 %)	0.77
Fluid overload, *n* (%)	17 (30.4 %)	9 (16.1 %)	0.12
Respiratory tract infection, *n* (%)	3 (5.4 %)	11 (19.6 %)	0.04
Unidentified factor, *n* (%)	10 (17.9 %)	14 (25.0)	0.49
Other, *n* (%)	8 (14.3 %)	7 (12.5 %)	>0.99
At admission			
Respiratory rate, cycles/min	32 ± 7	32 ± 7	0.77
Heart rate, beats/min	109 ± 28	96 ± 20	<0.01
Systolic blood pressure, mmHg	151 ± 35	157 ± 36	0.33
Glasgow coma scale, points	14.8 ± 0.7	14.0 ± 2.5	0.08
pH, units	7.40 ± 0.06	7.25 ± 0.10	<0.01
pH <7.35, *n* (%)	10 (18 %)	44 (79 %)	<0.01
PaO_2_, mmHg	87 ± 45	120 ± 80	<0.01
PaCO_2_, mmHg	36 ± 5	63 ± 16	<0.01
Bicarbonates, mmol/L	23.2 ± 3.8	29.0 ± 6.9	<0.01
N-terminal pro-BNP, ng/L	20720 ± 36085	23,003 ± 39,928	0.59
Troponin, μg/L	0.296 ± 1.053	0.207 ± 0.925	0.11
At initiation of NIV			
Respiratory rate, cycles/min	30 ± 7	30 ± 8	0.98
Heart rate, beats/min	105 ± 24	93 ± 20	0.01
Systolic blood pressure, mmHg	136 ± 25	144 ± 34	0.22
Altered consciousness (RASS<0), *n* (%)	2 (4 %)	11 (20 %)	0.02
FiO_2_, %	67 ± 24	60 ± 27	0.21
PEEP level, cm H_2_O	4.9 ± 1.7	4.6 ± 1.8	0.35
Pressure-support level, cm H_2_O	7.9 ± 1.8	10.0 ± 2.7	<0.01
Expiratory tidal volume, ml	604 ± 172	496 ± 125	<0.01
pH, units	7.42 ± 0.09	7.35 ± 0.08	<0.01
Minute ventilation, L/min	17.6 ± 6.0	14.5 ± 5.4	0.02
PaCO_2_, mmHg	37 ± 9	51 ± 13	<0.01
PaO_2_/FiO_2_, mmHg	247 ± 108	234 ± 88	0.68
Leaks or poor tolerance, *n* (%)	5/43 (19 %)	10/41 (15 %)	0.16
Patients’ outcome			
NIV duration, days	1.0 [1.0–2.0]	2.0 [1.0–3.0]	0.14
ICU Length of stay, days	4.0 [2.8–6.0]	4.0 [2.8–6.3]	0.97
Non-rapid favorable outcome, *n* (%)	13 (23.2 %)	18 (32.1 %)	0.40
Prolonged NIV (>48 h), *n* (%)	9 (16.1 %)	15 (26.8 %)	0.25
Intubation, *n* (%)	4 (7.1 %)	3 (5.4 %)	>0.99
In-ICU mortality, *n* (%)	2 (3.6 %)	2 (3.6 %)	>0.99

*Abbreviations*: *SAPS* simplified acute physiological score, *BMI* body mass index, *LVEF* left ventricular ejection fraction, *CPE* cardiogenic pulmonary edema, *NIV* non-invasive ventilation, *RASS* Richmond agitation-sedation scale, *PEEP* positive end-expiratory pressure, *ICU* intensive care unit

### Factors associated with poor outcome

**Fig. 2:**
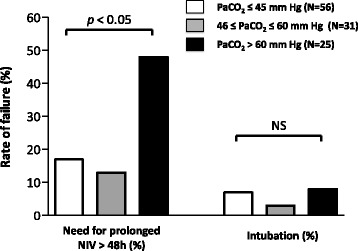
Rate of non-rapid favorable outcome, including the need for intubation or continuation of NIV for more than 48 h, according to the level of PaCO_2_ at admission. A higher proportion of patients with PaCO_2_ >60 mmHg (indicated by *black bars*) needed prolonged NIV beyond 48 h, whereas the rate of intubation was similar regardless of PaCO_2_ value at admission

**Fig. 3:**
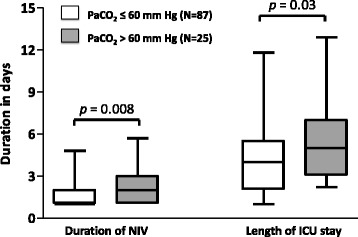
Box plots showing the median [25^th^–75^th^ percentiles] durations of NIV and of ICU length of stay according to the level of PaCO_2_ at admission (≤60 mmHg or >60 mmHg). The durations of NIV and of ICU length of stay were significantly longer by a median of 1 day in patients with PaCO_2_ >60 mmHg as compared to patients with PaCO_2_ ≤60 mmHg

Comparisons between patients who had a rapid favorable outcome and the others are given in Table 2. Although the proportion of hypercapnic patients at admission (PaCO_2_ >45 mmHg) was similar in the two groups, patients with non-rapid favorable outcome had more frequently severe hypercapnia defined as PaCO_2_ >60 mmHg (Table 2). After adjustment using logistic regression, the only independent factor associated with non-rapid favorable outcome was severe hypercapnia (PaCO_2_ >60 mmHg), at admission (OR = 4.15 [95 % CI 1.62–10.6]; *p* = 0.003). The need for intubation was uncommon and did not differ between patients with severe hypercapnia at admission and the others with a rate of only 8 % (2/25) vs. 6 % (5/87), *p* = 0.65, respectively. However, patients with severe hypercapnia were more likely to receive prolonged NIV than patients with no or moderate hypercapnia (Fig. 2). As shown Fig. 3, the durations of NIV and of ICU stay were both prolonged by a median of 1 day in patients with severe hypercapnia as compared to the others (2.0 days [1.0–3.0] vs. 1.0 [1.0–2.0], *p* = 0.003, and 5.0 days [3.0–7.0] vs. 4.0 [2.0–5.5], *p* = 0.03, respectively). Patients with severe hypercapnia at admission had lower pH values and a higher PaCO_2_ than the others patients 24 h after NIV initiation: (7.36 ± 0.08 vs. 7.46 ± 0.06, and 60 ± 15 mmHg vs. 38 ± 7, *p* < 0.001 for both).

**Table 2:** Comparison of patients according to outcome

	Rapid favorable outcome, *N* = 81	Non-favorable outcome, *N* = 31	*P* value
Characteristics of the patients			
Age, years	71.9 ± 13.1	76.7 ± 14.6	0.21
Male gender, *n* (%)	42 (52 %)	15 (48 %)	0.83
SAPS II, points	36.2 ± 11.8	41.9 ± 16.1	0.06
BMI, kg/m^2^	25.7 ± 5.7	26.3 ± 6.5	0.94
Smoker, *n* (%)	28 (35 %)	12 (39 %)	0.83
Underlying ischemic cardiopathy, *n* (%)	40 (49 %)	15 (48 %)	>0.99
LVEF, %	41 ± 14	42 ± 14	0.69
Reason for CPE			
Atrial fibrillation, *n* (%)	8 (9.9 %)	3 (9.7 %)	>0.99
Acute myocardial infarction, *n* (%)	8 (9.9 %)	0	0.10
Hypertensive crisis, *n* (%)	11 (13.6 %)	3 (9.7 %)	0.75
Overload, *n* (%)	21 (25.9 %)	5 (16.1 %)	0.33
Infection of respiratory tract, *n* (%)	8 (9.9 %)	6 (19.4 %)	0.21
Other, *n* (%)	8 (9.9 %)	7 (22.6 %)	0.12
Unidentified factor, *n* (%)	17 (21.0 %)	7 (22.6 %)	>0.99
At admission			
Respiratory rate, cycles/min	32 ± 7	32 ± 6	0.96
Heart rate, beats/min	102 ± 27	103 ± 19	0.69
Systolic blood pressure, mmHg	154 ± 37	153 ± 32	0.93
Glasgow coma scale, points	14.5 ± 1.9	14.3 ± 1.9	0.16
pH, units	7.33 ± 0.11	7.31 ± 0.11	0.27
PaO_2_, mmHg	100 ± 60	113 ± 81	0.64
PaCO_2_, mmHg	47 ± 15	55 ± 22	0.16
PaCO_2_ >45 mmHg, *n* (%)	38 (47 %)	18 (58 %)	0.40
PaCO_2_ >60 mmHg, *n* (%)	12 (15 %)	13 (42 %)	<0.01
Bicarbonates, mmol/L	25.3 ± 4.7	28.0 ± 8.8	0.25
N-terminal pro-BNP, ng/L	21,398 ± 37,463	23,115 ± 39,739	0.48
Troponin, μg/L	0.22 ± 0.88	0.32 ± 1.21	0.67
At initiation of NIV			
Heart rate, beats/min	100 ± 25	98 ± 19	0.70
Systolic blood pressure, mmHg	142 ± 32	136 ± 26	0.66
Altered consciousness (RASS <0), *n* (%)	8 (10 %)	5 (16 %)	0.51
Respiratory rate, cycles/min	30 ± 8	30 ± 6	0.92
Expiratory tidal volume, ml	568 ± 151	513 ± 114	0.047
Minute ventilation, L/min	16.4 ± 5.6	15.3 ± 6.4	0.25
Pressure-support level, cm H_2_O	8.2 ± 1.9	10.2 ± 2.9	<0.01
PEEP level, cm H_2_O	4.8 ± 1.7	4.7 ± 1.8	0.60
FiO_2_, %	66 ± 25	59 ± 27	0.27
pH, units	7.40 ± 0.09	7.36 ± 0.08	0.015
PaCO_2_, mmHg	41 ± 11	50 ± 16	0.017
PaCO_2_ >45 mmHg	11/71 (15 %)	16/31 (52 %)	<0.01
PaO_2_/FiO_2_, mmHg	243 ± 99	235 ± 98	0.67
PaO_2_/FiO_2_ ≤200 mmHg	27/71 (38 %)	10/31 (32 %)	0.66
Patients’ outcome			
NIV duration, days	1.0 [1.0–2.0]	3.0 [3.0–4.0]	<0.01
ICU Length of stay, days	3.0 [2.0–5.0]	7.0 [5.0–9.5]	<0.01

*Abbreviations*: *SAPS* simplified acute physiological score, *BMI* body mass index, *LVEF* left ventricular ejection fraction, *CPE* cardiogenic pulmonary edema, *NIV* non-invasive ventilation, *RASS* Richmond agitation-sedation scale, *PEEP* positive end-expiratory pressure, *ICU* intensive care unit

In patients with a history of smoking or with a BMI >30 kg/m^2^, the rate of non-rapid favorable outcome was similar to that observed in patients without none of these two risk factors for chronic lung disease, 28 % (15/53) vs. 27 % (16/59), respectively, *p* > 0.99.

## Discussion

We assessed the role of hypercapnia on outcome of patients treated with NIV for severe CPE and having no identified underlying chronic lung disease. We found that half of the patients had hypercapnia at admission. Hypercapnic patients were older, more frequently obese, had more frequently infection of respiratory tract, and had lower tidal volumes despite higher pressure-support levels. Outcome was similar between hypercapnic and non-hypercapnic patients. However, patients with severe hypercapnia (PaCO_2_ >60 mmHg) at admission required a longer duration of NIV without increase in intubation rate.

### Incidence and factors associated to hypercapnia

Our study shows that half of the patients with severe CPE had hypercapnia upon ICU admission. Although no previous studies has systematically excluded all patients with any underlying chronic lung disease as we did, this 50 % rate is in keeping with the literature reporting a 40 to 50 % proportion of hypercapnic patients among those admitted with CPE [7, 8, 10–12, 14]. Despite higher pressure-support levels during NIV, hypercapnic patients had lower tidal volumes and appeared therefore unable to increase minute ventilation to compensate for hypercapnia. A small tidal volume suggests a weak patient effort or an altered compliance of the respiratory system. In the absence of CLD, hypercapnic patients were older and more frequently obese than non-hypercapnic patients. Whereas elderly patients might rapidly suffer from muscle weakness and diaphragmatic fatigue and therefore generate weak inspiratory efforts, obese patients have marked reduction in compliance in respiratory system due to increased abdominal pressure and stiff thoracic cage, reducing tidal volumes and promoting hypercapnia. These two factors, associated to extensive airway collapse and/or obstruction by alveolar edema fluid, can promote hypercapnia and may concur to the inability to maintain adequate alveolar ventilation.

### Role of hypercapnia on outcome

We found that hypercapnia upon admission did not influence outcome. However, severe hypercapnia (PaCO_2_ >60 mmHg) at admission was associated with a non-rapid favorable outcome, although the rate of intubation did not differ and the only difference between patients with severe hypercapnia and the others was the need for longer duration of NIV and of ICU stay by only 1 day. Several studies found an increased rate of intubation in hypercapnic patients as compared to non-hypercapnic patients [6, 14]. Although all patients received NIV, Nouira and colleagues found a 20 % intubation rate in hypercapnic patients vs. only 4 % in the others [6]. The impact of hypercapnia on outcome must be analyzed with caution since most of studies did not exclude patients with CLD which may confound the interpretation.

NIV can be delivered using continuous positive airway pressure (CPAP) or using bi-level positive airway pressure with pressure-support (PS). Whatever the ventilation strategy used, positive airway pressure improves cardiac performance by decreasing preload and afterload [28], and improves respiratory function by decreasing work of breathing [29], resulting a rapid improvement in oxygenation. However, PSV is particularly effective in hypercapnic patients, and several studies found that respiratory distress improved more rapidly using PSV than using CPAP [2, 6]. Contrarily to the above-mentioned studies, one study has suggested that hypercapnic patients treated using PSV may have a better outcome than non-hypercapnic patients [7]. However, in that study, the rate of intubation was as expected in hypercapnic patients (6 %), whereas it was exceedingly high (34 %) in non-hypercapnic patients. With the use of PSV, we found no difference in intubation rate between hypercapnic and non-hypercapnic patients, in keeping with another study showing that acidemia whether respiratory or metabolic upon admission had no impact on outcome [10]. We also found no difference in terms of blood pressure between patients with non-rapid favorable outcome and the others, whereas a lower systolic arterial blood pressure had been associated to worst outcome in a previous study [30].

### Clinical implications

Although hypercapnia usually defined as PaCO_2_ >45 mmHg at admission had no influence on outcome, clinicians should be aware that patients with CPE presenting with severe hypercapnia (PaCO_2_ >60 mmHg) may require more prolonged NIV than those with no or moderate hypercapnia. Whereas the rate of intubation in patients with obesity-hypoventilation is close to that reported in CPE [31], i.e., below 10 %, several controlled trials reported a rate of intubation between 20 and 30 % in COPD patients treated by NIV [16, 32, 33]. Therefore, an underlying CLD or obstructive sleep apneas should be systematically suspected in patients with CPE requiring intubation or prolonged NIV.

### Limitations

Our study has several limitations. First, it was conducted in a single unit with a long-standing experience in the practice of NIV, and therefore our results may not be applicable to other centers. Second, some hypercapnic patients included may still have underlying CLD leading to potential alveolar hypoventilation. However, among hypercapnic patients, most of them (57 %) had never smoked making it unlikely to have COPD while 69 % of them had a body mass index below 30 kg/cm^2^ excluding obesity-hypoventilation syndrome. Moreover, we put substantial efforts during this study in identifying patients having underlying CLD and sleep apneas. We arbitrarily defined non-rapid favorable outcome as the need for NIV longer than 48 h which identified the worst quartile of patients needing longer duration of NIV. Although no criterion is established to define a rapid favorable outcome, CPE is usually rapidly reversible the first 24 h of therapy. Lastly, given that intubation was an uncommon event, we could not analyze the independent risk factors of poor outcome and consequently the real impact of hypercapnia on outcome. Although all our patients with acute myocardial infarction had a rapid favorable outcome, the number of patients was too small to highlight a potential difference.

## Conclusions

Hypercapnia occurred in half of the patients among the most severe admitted to the ICU for CPE, even after excluding all those with any underlying CLD. Hypercapnic patients were older and were more frequently obese. The rates of intubation or prolonged NIV for more than 48 h were similar between hypercapnic and non-hypercapnic patients. However, patients with severe hypercapnia required longer durations of NIV and ICU stay.

## Abbreviations

CLD: Chronic lung disease
COPD: Chronic obstructive pulmonary disease
CPAP: Continuous positive airway pressure
CPE: Cardiogenic pulmonary edema
ICU: Intensive care unit
OSA: Obstructive sleep apnea
NIV: Non-invasive ventilation
PEEP: Positive end-expiratory pressure
